# Investigating the development of diarrhoea through gene expression analysis in sheep genetically resistant to gastrointestinal helminth infection

**DOI:** 10.1038/s41598-022-06001-4

**Published:** 2022-02-09

**Authors:** Shamshad Ul Hassan, Eng Guan Chua, Erwin A. Paz, Parwinder Kaur, Chin Yen Tay, Johan C. Greeff, Shimin Liu, Graeme B. Martin

**Affiliations:** 1 UWA School of Agriculture and Environment, The University of Western Australia, Crawley, WA 6009 Australia; 2 Helicobacter Research Laboratory, The Marshall Centre for Infectious Disease Research and Training, School of Biomedical Sciences, University of Western Australia, Perth, WA Australia; 3 Department of Primary Industries and Regional Development, Western Australia, 3 Baron Hay Court, South Perth, WA 6151 Australia

**Keywords:** Computational biology and bioinformatics, Immunology, Molecular biology

## Abstract

Gastrointestinal helminths infect livestock causing health problems including severe diarrhoea. To explore the underlying biological mechanisms relating to development and control of diarrhoea, we compared 4 sheep that were susceptible to development of diarrhoea with 4 sheep that were diarrhoea-resistant. Transcriptomes in the tissues where the parasites were located were analyzed using RNASeq. By considering low-diarrhoea sheep as control, we identified 114 genes that were down-regulated and 552 genes that were up-regulated genes in the high-diarrhoea phenotype. Functional analysis of DEGs and PPI sub-network analysis showed that down-regulated genes in the high-diarrhoea phenotype were linked to biological processes and pathways that include suppression of ‘antigen processing and presentation’, ‘immune response’, and a list of biological functional terms related to ‘suppression in immune tolerance’. On the other hand, up-regulated genes in the high-diarrhoea phenotype probably contribute to repair processes associated with tissue damage, including ‘extracellular matrix organization’, ‘collagen fibril organization’, ‘tissue morphogenesis’, ‘circulatory system development’, ‘morphogenesis of an epithelium’, and ‘focal adhesion’. The genes with important roles in the responses to helminth infection could be targeted in breeding programs to prevent diarrhoea.

## Introduction

The sheep is globally relevant to production of milk, meat, and wool for human consumption. These industries contribute significantly to the world economy and are particularly important for Australia, one of the largest sheep-producing countries in the world (www.fao.org)^1^. One major challenge is the disease caused by parasites^2^. For example, in south-western Australia, owing to its Mediterranean climate, the winter rainfall season provides conditions that allow the larvae of gastrointestinal helminths to flourish on pasture, and thus infect grazing sheep. The major helminth species in this region, *Teladorsagia circumcinta* and *Trichostrongylus colubriformis*^3^, are responsible for significant losses in productivity and they also cause diarrhoea that predisposes the host to flystrike, a serious concern for animal welfare.

As licensed vaccines are not available for *T. circumcinta* or *T. colubriformis*^4,5^, the dominant approach for many decades has been to use anthelmintic medications^6^. However, the helminths are becoming increasingly resistant to them^4^. Moreover, there is an increase in consumer demand for residue-free and chemical-free products, placing extra pressure on this option^7^. The most promising long-term solution seems to be genetic selection of sheep that resist helminths^8^, a path taken in 1988 by the Department of Primary Industries and Regional Development, Western Australia. The outcome has been the ‘Rylington’ flock of Merino sheep that are highly resistant to helminths, with very low faecal egg counts (FEC) for the dominant *Teladorsagia* and *Trichostrongylus* species^8–10^. However, breeding for helminth resistance appears to have led to an increase in susceptibility to diarrhoea^11,12^. To further address the diarrhoea problem, a ‘breech-strike flock’ was developed by selecting for the low-diarrhoea phenotype. In both flocks, diarrhoea has been assessed by faecal consistency score (or the more colloquial ‘dag’ score), a subjective measure of the amount of faecal matter accumulated around the anus that is a heritable trait^13^.

Helminths evoke both adaptive and innate immune responses^14^. The adaptive (long-term) response is initiated by the binding of helminth antigen to CD4^+^ T cells through molecules of the major histocompatibility complex class II (MHC-II), eliciting a Th2 antibody-mediated response. This leads to the secretion of Th2-type cytokines (IL-13, IL-5, IL-4), infiltration of mast cells and eosinophils, and production of antibodies (IgE and/or IgA^15,16^). The innate immune response, on the other hand, comprises the physical barriers (gut mucus layer, defensins, trefoil factors, enteric muscle contractility)^17,18^, pattern recognition receptors (C-type lectin receptors and Toll-like receptors expressed by antigen-presenting immune cells in tissues)^19^, proinflammatory and cytotoxic cells (eosinophils, mast cells)^20^, and various chemo-attractants (such as IL-5, eotaxin family of chemokines CCL26, CCL24, CCL11)^21^. These innate responses act to expel the helminths from the gastrointestinal tract, and often involve diarrhoea. However, diarrhoea can also be caused by damage to gastrointestinal mucosa by the helminths, increased gut permeability, changes in gut motility^22,23^ and, in some cases, ‘hypersensitivity scouring/diarrhoea’– a heightened immune response to the larvae^12,24,25^. A number of studies have linked susceptibility to helminths in sheep to a Th1-mediated immune response, involving Th1-type cytokines (IL-12, INF-γ) production and proliferation of CD8^+^ cytotoxic T cells^26^. The diarrhoea is thus a complex problem and does not depend upon any single factor^25^.

To resolve the problem, we need to go beyond the measurements of environmental factors and phenotypic aspects (FEC, diarrhoea score), and develop an understanding of the biological mechanisms that underlay the helminth-diarrhoea relationship. Most of the physiological mechanisms are controlled by normal gene expression and most of the pathological processes can be explained by disrupted gene expression^27^. Therefore, analysis of gene expression and the associated molecular pathways should be useful for studying and grouping genes that are responsible for a phenotype. Such studies have already provided promising gene candidates with respect to helminth resistance and diarrhoea control^28^. For example, IL-13, IL-4 and IL-5, the humoral immune response, protein synthesis and the inflammatory response, were all found to be central to a phenotype for resistance to *T. circumcinta* infection in the abomasal lymph node transcriptome^29^. In another study of abomasal mucosa and abomasal lymph nodes, ITLN2*,* CLAC1*,* galectins, the PPARG signaling pathway and the cytokine-mediated immune response, were associated with resistance to *T. circumcinta* infection in sheep^28^. Moreover, resistance to helminths has been associated with allelic forms of candidate genes, including those for MHC Class I and II^30^ and MHC-DRB^31^.

So far, the genetic selection of animals has relied on two phenotypic traits—FEC (described above) and an indictor trait for diarrhoea^32^—and has not explored molecular markers that could be more reliable for high resolution selection^33^. The three ways to identify such markers include genome-wide association studies (GWAS), the mapping of quantitative trait loci (QTL) and potential gene expression analysis (e.g., RNASeq), with the latter being more practical for evaluating links between variation in gene expression and phenotypic traits^29,34^. Generally, the relationship between helminths and diarrhoea has been considered an outcome of the infection^35^ but it is clear that the two can be independent, as shown by studies in sheep where the level of diarrhoea is often not related to FEC, total helminth burden, or the number of infective larvae administered^36,37,38^. We have therefore studied animals that are susceptible and not susceptible to development of diarrhoea. In addition, most studies on the molecular links to low- and high-diarrhoea phenotypes have been studied under controlled conditions, using tissues other than the duodenum (the site of infection). For example, in our previous study of Merino sheep that were genetically prone or not prone to develop diarrhoea, and genetically resistant and susceptible to helminth infection, in a winter rainfall region, we assessed haematology (haemoglobin, red blood cell count, packed cell volume, white blood cell count) and found no significant differences among the groups^39^. However, haematology profiles are still useful as indicators of *Haemonchus contortus* infestations in summer rainfall regions^40^.

In the current study, we therefore investigated the molecular mechanisms and pathways in the duodenum through differential gene expression analysis in sheep that were susceptible or resistant to the development of diarrhoea. We tested whether tendency for diarrhoea is caused by up-regulation of genes related to the innate and adaptive immune responses. Specifically, we tested the hypothesis that diarrhoea is caused by up-regulation of genes related to the inflammatory/hypersensitive immune response.

## Materials and methods

### Animals, location, experimental design, and ethics statement

All of the experimental work was approved by the Animal Ethics Committee of the Department of Primary Industries and Regional Development, Western Australia (AEC No.17-1-02). Lambs were selected on the basis of Australian Sheep Breeding Values (ASBVs) and sourced from the flocks that had been maintained at the Katanning Research Facility (Western Australia) since 2015 as part of a large, long-term study addressing the helminth-diarrhoea-flystrike complex. The Katanning facility is located at an elevation 300 m, longitude 117.5 5°E and latitude of 33.7°S, with an average yearly rainfall of 480 mm, predominantly in winter, typical of a Mediterranean climatic zone.

The experiment started with a total of 986 lambs. Faeces were sampled from them all at weaning (100 days of age) when they were also given a broad-spectrum anthelmintic (monepantel; 1 mL/10 kg body weight) orally in November 2016. For all lambs, diarrhoea scores at weaning and completed pedigrees had been submitted to Sheep Genetics (www.sheepgenetics.org.au) for the estimation of Australian Sheep Breeding Values (ASBVs). Individuals with extreme high and low breeding values for diarrhoea score were then identified, and 100 males and 100 females were selected.

From November 2016 until September 2017, the lambs were kept in single-sex flocks in two fields, but otherwise managed similarly, where they grazed an annual grass clover pasture with capeweed (*Arctotheca calendula*). The stocking rate was kept at 10 lambs per hectare. The pasture had not been grazed for 4 months before the experiment began in March 2017, allowing the lambs to acquire a natural helminth infection under natural conditions. The pastures were kept green by unusual rain in February and subsequent rainfalls (in March, April and May), so the sheep had access to green fodder throughout the experiment, from February until the end in September, in contrast to normal season when green fodder does not become available until May. The dominant parasite species at the start of experiment were *Teladorsagia* spp., then *Trichostrongylus* spp., followed by *Chabertia ovina* and *Oesophagostomum venulosum*, and a small fraction of *Haemonchus contortus*. This species composition changed by the end of experiment as *Trichostrongylus* spp. became dominant, followed by *C. ovina*, *O*. *venulosum*, *Teladorsagia* spp. and a small fraction of *H. contortus*^39^.

**Figure 1:**
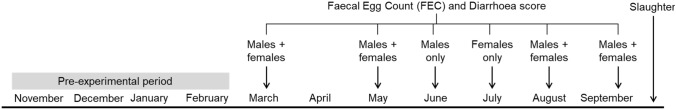
Schema of the experimental protocol, showing timing of measurements (FEC and diarrhoea score sheep) from March (autumn) through to September (spring). For the 4-month pre-experimental period leading up the first measurement, the pasture was not used for grazing to avoid contamination with helminth eggs. In this environment, helminth infection is usually associated with the onset of winter rains in April–May.

In March, May June, August, and September (Fig. 1), all 200 lambs were scored for diarrhoea, using a scale of 1 to 5 (low to high) based on the amount of faecal material accumulated on the hind quarters, between the anus and the hock joint. At the same time, FEC was measured. Note that FEC; ≥ 500 helminth eggs per gram faeces is considered as high under these conditions. In September 2017, 18 diarrhoea-resistant (low-diarrhoea; LD) and 20 diarrhoea-susceptible (high-diarrhoea; HD) lambs were slaughtered for the collection of duodenal tissues. From these 38 lambs, four replicates from each of the two groups were selected for the present study. Each HD or LD consisted of two males and two females. The tissues were kept in RNAlater solution (Sigma-Aldrich, St. Louis, United States) and stored as per manufacturer’s instructions at − 80 °C before RNA extraction.

### RNA extraction, library preparation, quality control and sequencing

From each of the eight selected lambs, approximately 30 mg tissue was sampled from the duodenum. Total RNA was extracted using the RNeasy mini plus kit (Qiagen, Hilden, Germany) according to manufacturer’s instructions with minor modifications. Briefly, after tissue homogenization in lysis buffer, tubes were centrifuged at 14,000×*g* for 3 min. The supernatant was loaded onto the gDNA removal column and centrifuged at 10,000×*g* for one min, following which an equal volume of 70% (v/v) ethanol was added to the flow-through fraction and mixed by pipetting. The mixture was loaded onto the RNA binding column and centrifuged at 10,000×*g* for 15 s. The column was washed once with RW1 wash buffer, prior to adding 80 µL of DNase-I (0.34 Kunitz/µL) (Qiagen, Hilden, Germany) for 15 min to ensure complete gDNA removal. Subsequently, the column was washed again with RW1 buffer and twice with RPE buffer before eluting the RNA sample with RNAse free water.

The RNA content was quantified with a Qubit fluorometer using an RNA-BR kit and the purity was checked using NanoDrop. Quality was checked on a 2% agarose gel by assessing the 28S/18S ribosomal RNA ratio. Only samples with an OD 260/280 ratio of greater than 2 and a 260/230 ratio of 1.8 or greater were sent to BGI Genomics in Hong Kong for library preparation and sequencing. The sequencing libraries prepared from RNA samples with RIN (RNA integrity number) values ≥ 7 were sequenced using the 100 bp paired-end chemistry on DNBseq™ platform, with a depth of ≥ 20 million reads/sample.

### Data processing

The quality of the reads was checked by FastQC and they all scored above 30. The reads were then aligned with the reference genome (Oar_rambouillet_v1.0) using STAR (v2.7.3a)^41^ with default parameters. After alignment, the featureCounts function in the Subread (version 2.0.0)^42^ software was used to enumerate the number of reads mapping to each gene using the following parameters; *-p* *–primary -t gene.* Differential gene expression analysis was aided by the DESeq2 R package, using the low-diarrhoea samples as the reference group^43^. A differentially expressed gene (DEG) was defined as a gene with a false discovery rate (FDR) < 0.05 and a log2 fold change > 1. To estimate and visualize the variations between samples, a principal component analysis (PCA) was performed on the DESeq2-normalised count data after regularized log transformation.

### Functional enrichment analysis

Functional enrichment analysis using the up-regulated and down-regulated DEGs was performed using the web-based tool, DAVID (The Database for Annotation, Visualization and Integrated Discovery)^44^. Additionally, the clusterProfiler (v3.18.1) R package was used for Gene Set Enrichment Analysis (GSEA) with org.Bt.eg.db (*Bos taurus*) being the source of annotation as the genome wide annotation list for sheep was not available. Significant Gene Ontology (GO) terms for biological processes and KEGG pathways with an FDR < 0.05 are reported.

To highlight functional interactions among the DEGs, the web-based Search Tool for the Retrieval of Interacting Genes/Proteins (STRING)^45^ database (v11), was used with the following options selected: ‘disconnected nodes hidden’; ‘interactions discarded with a confidence score < 0.4’; ‘no more than 5 interactors to show in 1st and 2nd shell’. Gene sub-networks that each contain at least five genes and a *p*-value < 0.05 were generated in Cytoscape (v 3.7.2) using the ClusterONE plugin that clusters genes by functional relevance. Enrichr, another web-based tool, was used to study the biological functions and pathways associated with genes in the sub-network analysis^46^.

### Ethics declarations

The authors confirm that the study was carried out in compliance with the ARRIVE guidelines. All animal experimentation was approved by the Animal Ethics Committee of the WA Department of Primary Industry and Rural Development, under the guidelines of the National Health and Medical Research Council’s Australian Code of Practice for the Care and Use of Animals for Scientific Purposes (approval no: AEC 17-1-02).

## Results

### Diarrhoea scores and FEC

The groups differed significantly for diarrhoea score (*p*-value = 0.02) but not for FEC (*p*-value = 0.85). The difference in diarrhoea score, along with the ASBV data used to select the animals studied, supports the use of gene expression analysis to investigate the genes associated with the tendency to develop diarrhoea.

### RNA-Seq data description and PCA outcome

**Figure 2:**
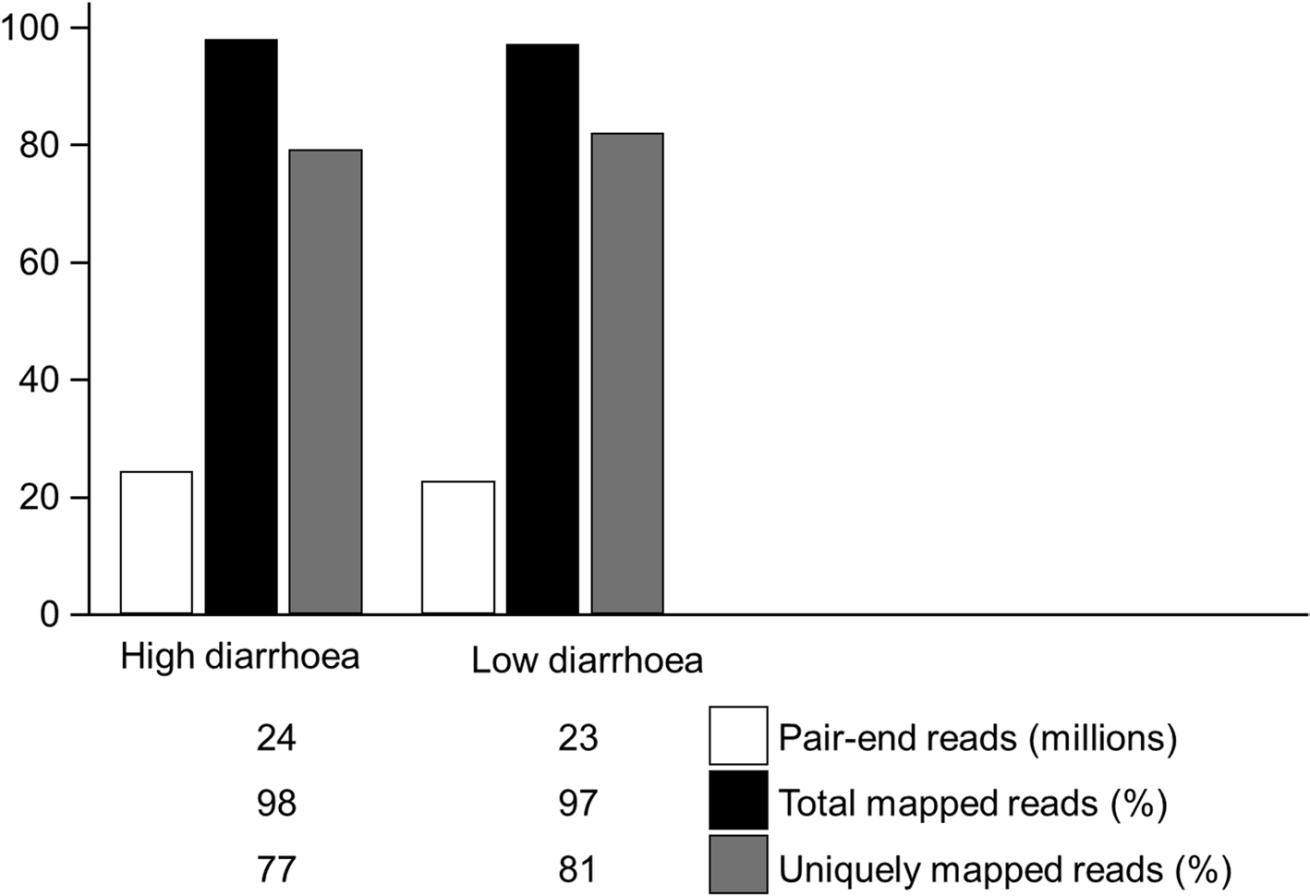
The mapping statistics for the RNASeq data. Each bar displays the mean total number of paired-end reads generated (white), the percentage of total reads successfully aligned against the reference genome (black), and the percentage of uniquely mapped reads (grey).

**Figure 3:**
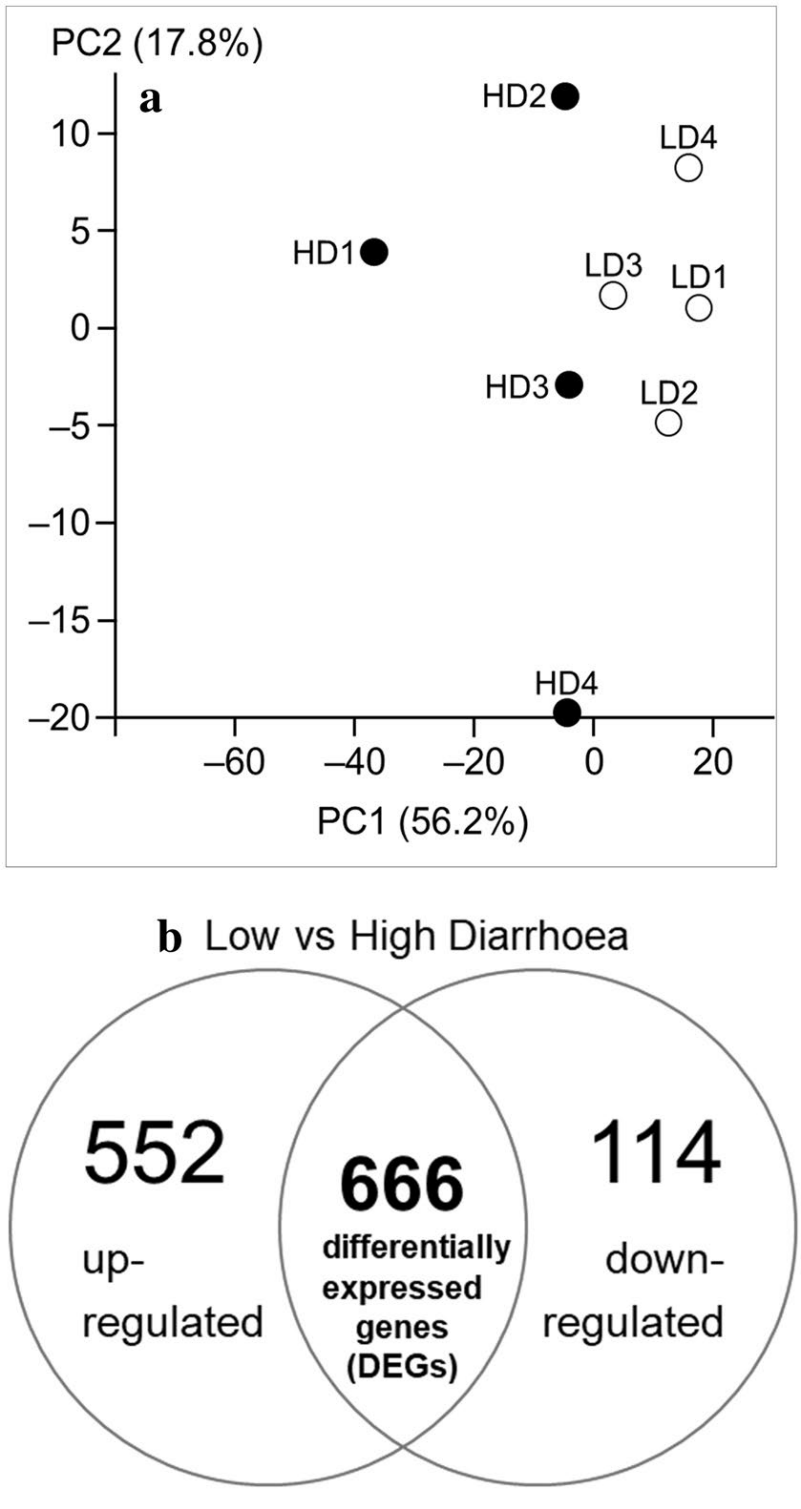
(**a**) PCA plot showing differences between HD and LD in gene expression. The PC1 and PC2 axes represent 56.2% and 17.8% of the total variance, respectively. (**b**) Venn-diagram presenting the total number of differentially expressed genes (DEGs), and the numbers of up- and down-regulated DEGs.

In this study, a total of 187,757,422 paired-end reads were generated, ranging from 21,654,075 to 25,460,771 paired-end reads per sample, with a minimum of 21,048,774 reads were successfully mapped against reference genome. The average unique mapping rate was 77% for the HD group and 81% for the LD group (Fig. 2). Next, our PCA plot (Fig. 3a) showed a tight clustering of the LD samples, whereas the HD samples were irregularly distributed, with PC1 representing 56.2% of the total variance and PC2 representing 17.8% of the total variance, indicating that there are inherent gene expression differences between the LD and HD duodenal samples.

### Differential gene expression and functional enrichment analyses

**Figure 4:**
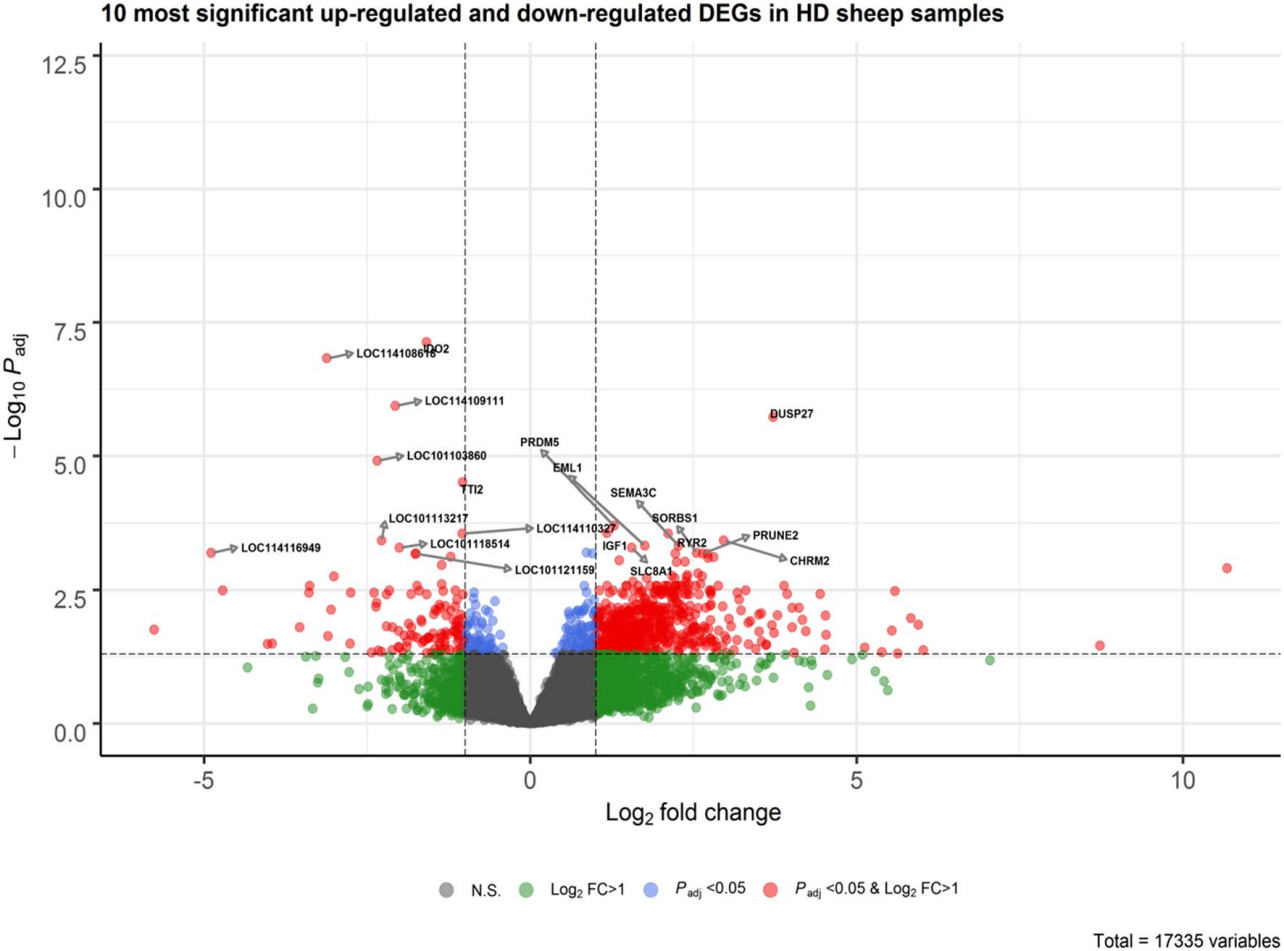
Volcano plot showing the 10 most significant up-regulated and down-regulated DEGs in HD sheep duodenal samples.

In the HD sheep samples, we identified 666 DEGs, of which 114 were down-regulated and 552 were up-regulated (Fig. 3b). For the LD samples, the control group in this study, the list of identified DEGs is presented in Supplementary File S1. A heatmap illustrating the top 100 most significant DEGs is presented in Supplementary Fig. S1. The 10 most significant up-regulated and down-regulated DEGs are shown in Fig. 4. Among the up-regulated DEGs were IGF-I, SEMA3C, EML1, DUSP27, PRDM5, SLC8A1, RYR2, SORBS1, PRUNE2 and CHRM2. Among the down-regulated DEGs were IDO2, TTI2, and several genes with functions that are yet to be determined.

**Figure 5:**
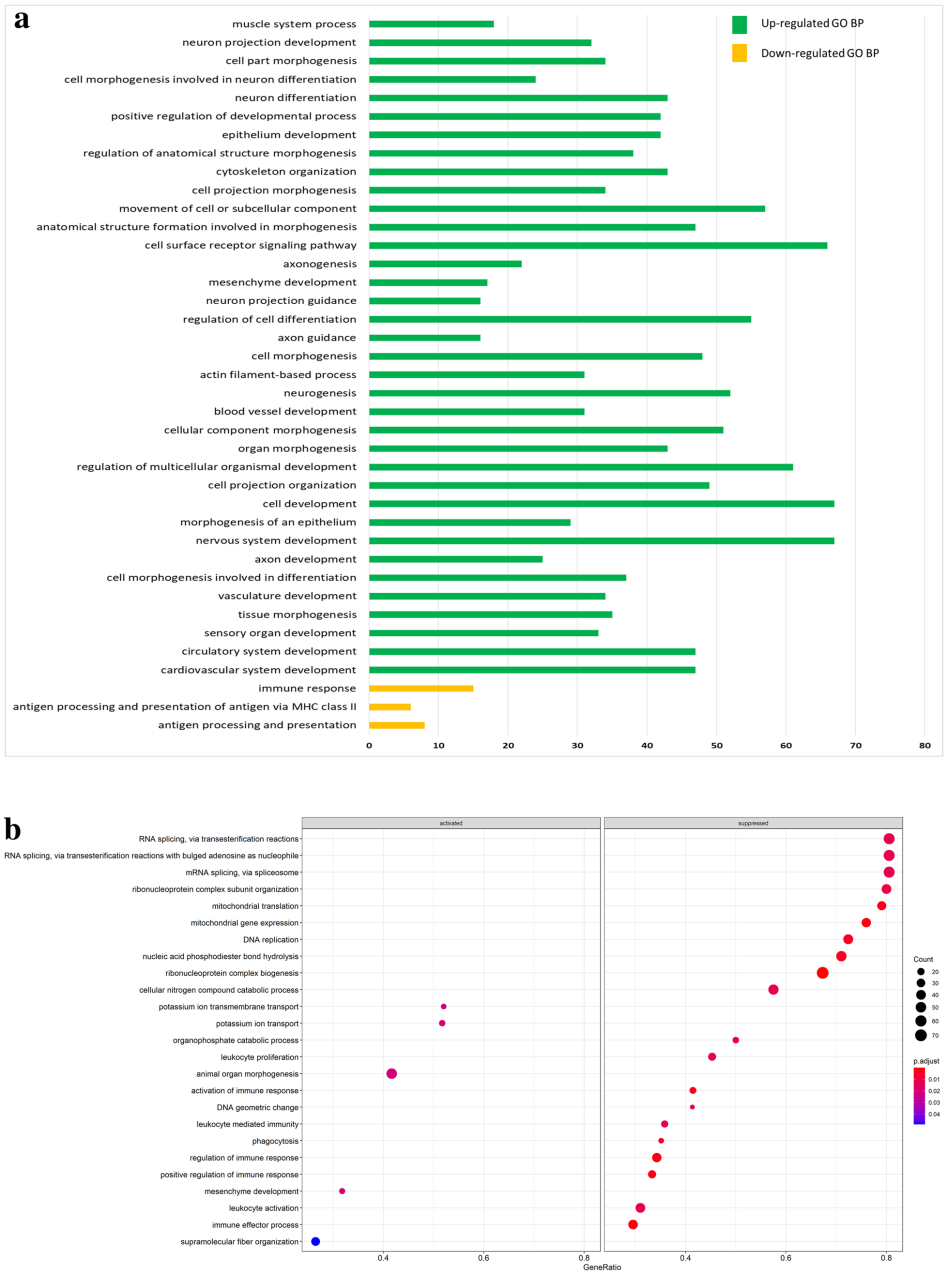
Functional enrichment analysis for GO biological process terms in the HD samples. (**a**) Analysis with DAVID using the up-regulated DEGs and down-regulated DEGs as the input data; the values on the x-axis represent the number of genes associated with each significant GO biological process term. (**b**) Analysis with GSEA utilizing the entire DESeq2-normalised dataset; the top 20 significantly enriched GO biological process terms are shown.

**Figure 6:**
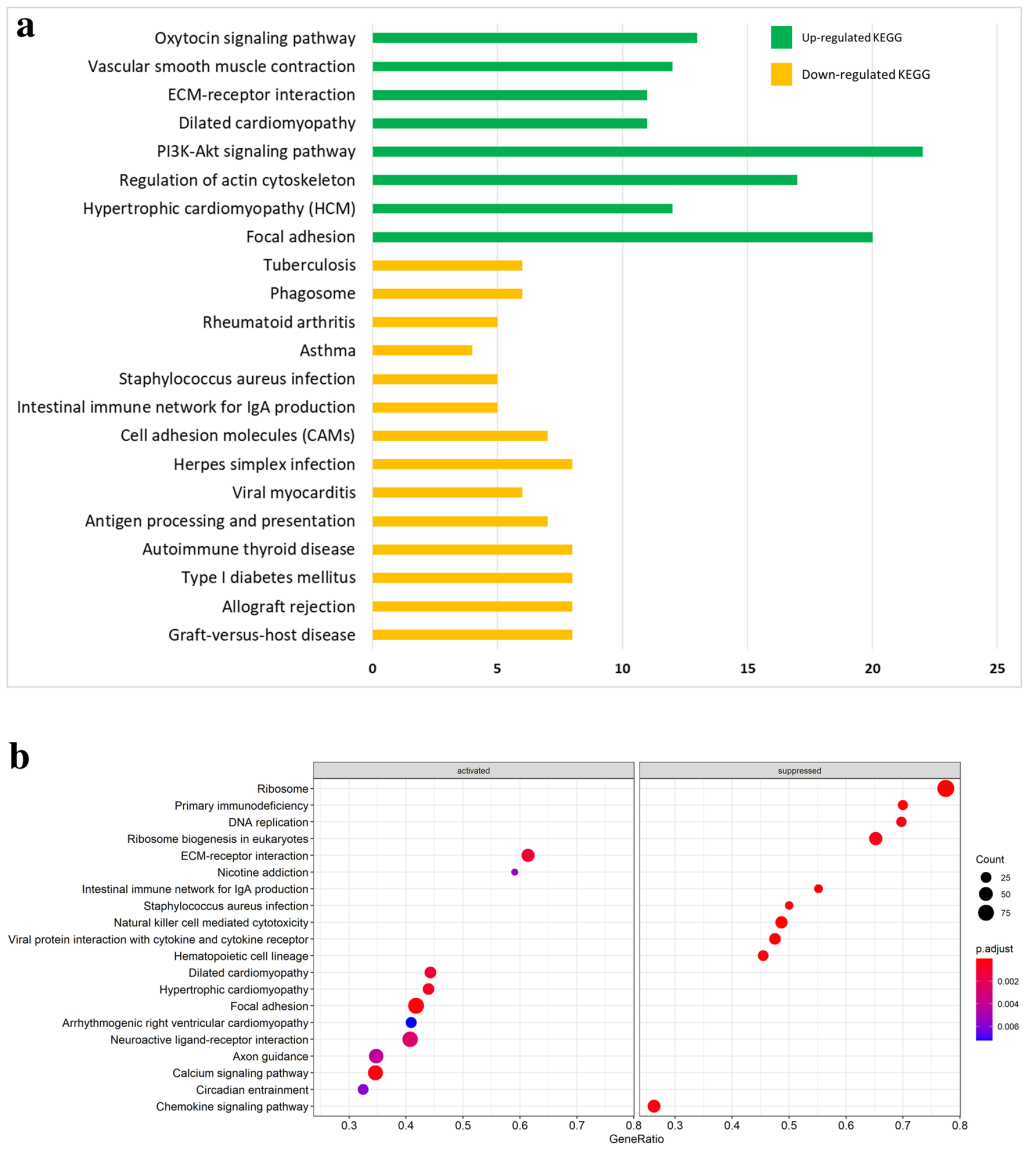
Functional enrichment analysis for KEGG pathways in the HD samples. (**a**) Analysis with DAVID using the up-regulated DEGs and down-regulated DEGs as the input data; the values on the x-axis represent the number of genes associated with each significant KEGG pathway. (**b**) Analysis with GSEA utilizing the entire DESeq2-normalised dataset; the top 20 significantly enriched KEGG pathways are shown in this figure.

Functional enrichment analyses, for identification of significantly enriched GO biological process terms and KEGG pathways, were performed using DAVID and the GSEA approach available in clusterProfiler. Importantly, the enrichment outcomes from both methods were similar. For the GO terms significantly enriched in the HD samples, both methods reported the up-regulation of ‘mesenchyme development’ and ‘organ development’, and the down-regulation of ‘immune response’-related biological processes (Fig. 5). The enrichment analysis based on DAVID revealed 13 significant KEGG pathways and the analysis GSEA revealed 63 significant KEGG pathways. The up-regulated KEGG pathways found in both analyses included ‘focal adhesion’, ‘PI3K-Akt signaling pathway’ ‘hypertrophic cardiomyopathy’, ‘dilated cardiomyopathy’, ‘ECM-receptor interaction’, ‘vascular smooth muscle contraction’ and ‘oxytocin signaling pathway’ (Fig. 6). The common down-regulated KEGG pathways comprised ‘allograft rejection’ and ‘antigen processing and presentation’. It is important to note that on GSEA analysis showed significant suppression of the pathway for ‘intestinal immune network for IgA production’ in the HD samples.

### PPI network and sub-network analysis

The DEGs were further annotated using the online STRING database to determine the relationships among them and how they interact to form protein–protein interaction (PPI) networks. In the HD samples, 74 of 114 down-regulated genes and 481 of 552 up-regulated genes were annotated as significant (PPI enrichment *p*-value < 0.001). The up-regulated genes formed only one significant subnetwork (SN) and were mostly associated with GO biological processes for ‘vesicle organization and vesicle-mediated transport’ and ‘calcium ion-dependent exocytosis’, and KEGG pathways for ‘synaptic vesicle cycle’, ‘SNARE interactions in vesicular transport’ and ‘insulin secretion’. The subnetwork plot of the up-regulated genes is presented in Supplementary Fig. S2.

**Figure 7:**
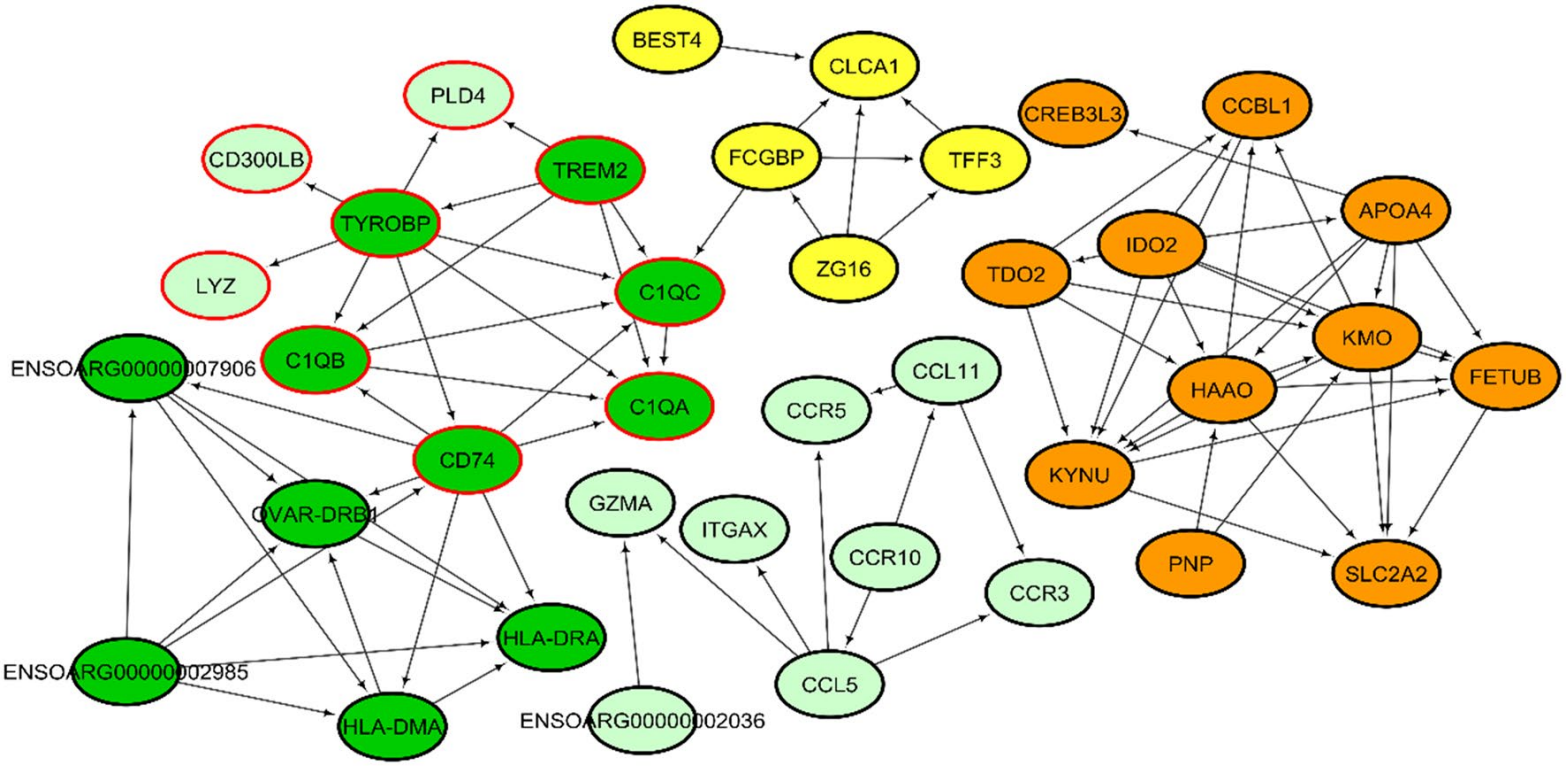
Protein–protein interaction (PPI) networks associated with down-regulated DEGs derived from STRING with subnetworks (SN) generated using the Cytoscape ClusterONE plugin. SN1 (dark green nodes); SN2 (orange nodes); SN3 (red bordered nodes); SN4 (yellow nodes); SN5 (blue bordered nodes).

The down-regulated genes were clustered into five significant SNs, as shown in Fig. 7. The genes in SN1 were significantly associated with biological processes linked to ‘antigen processing and presentation of exogenous peptide antigen via MHC class II’, ‘regulation of complement activation’ and ‘regulation of humoral immune response’. The KEGG pathways associated with SN1 included ‘antigen processing and presentation’, ‘intestinal immune network for IgA production’ and ‘complement and coagulation cascades’. For SN2, the genes were significantly linked to the KEGG pathway ‘tryptophan metabolism’. The full list of biological processes and KEGG pathways associated with each SN cluster are presented in Supplementary File S2.

## Discussion

The molecular processes and pathways that are activated by worm infection, and apparently influenced by genetic selection, differ clearly between sheep that are susceptible (HD) and resistant (LD) to the development of diarrhoea. The HD sheep tend to keep intact the physical barriers and promote tissue repair in an attempt to elicit diarrhoea to expel the helminths, whereas the LD sheep tackle the diarrhoea through innate and adaptive (Th2-mediated) immune responses (activated in low-diarrhoea and suppressed in high-diarrhoea). Following a natural helminth infection, a number of DEGs were up-regulated in HD duodenum that are related to repair mechanisms, whereas the down-regulated DEGs were mostly linked with a required immune response, not a hypersensitive immune response. These down-regulated DEGs were up-regulated in LD duodenum, explaining why fewer but more specific genes are up-regulated in specific biological processes and pathways that control diarrhoea. While some aspects of these issues could be better explored in a fully controlled laboratory experiment, the field study that we used offered an opportunity to select extreme genotypes from a large number of animals that had taken decades to develop, with outcomes that are relevant to real-world sheep management. Despite the limitations, we were able to reveal clear and strong relationships related to diarrhoea that are consistent with the literature and with the theoretical basis of immune responses to helminth infection.

**Figure 8:**
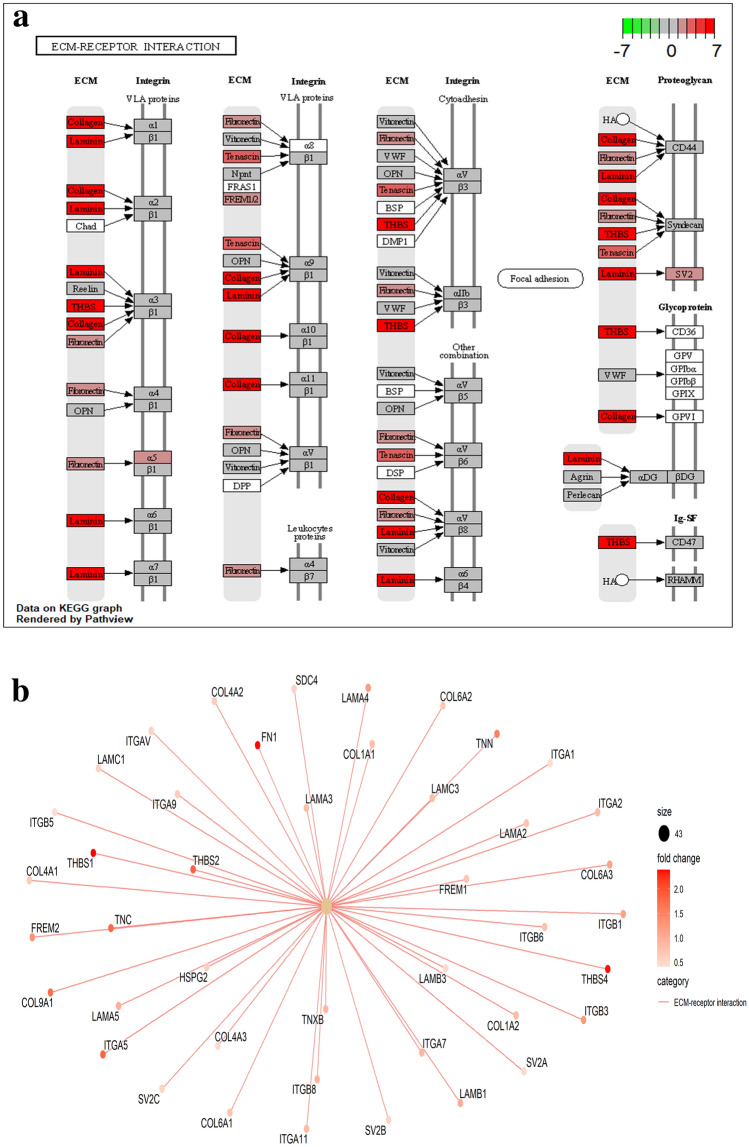
(**a**) The ECM-receptor interaction^47^, an important KEGG pathway, indicated by up-regulated genes in the high diarrhoea group. (**b**) A category Netplot (CNET) showing the relationships among genes in ECM-receptor interaction pathway.

In HD sheep, the biological processes and pathways associated with up-regulated genes indicate activation of repair mechanisms rather than activation of the immune system, specifically ‘tissue morphogenesis’, ‘focal adhesion’, ‘ECM-receptor interaction’, ‘cardiovascular system development’, ‘sensory organ development’, ‘vasculature development’, ‘vascular smooth muscle contraction’ and ‘PI3K-Akt signaling pathway’. Tissue morphogenesis is an important aspect not only during embryonic morphogenesis but during tissue repair. The response to tissue injury is a dynamic process, involving biochemical signals and mechanical forces leading to the formation of a fibrotic scar. The extracellular matrix (Fig. 8) links to the intracellular cytoskeleton through ‘focal adhesions’, a collection of multiple-protein-complexes playing a vital role in the proliferation of initial mechanical signals into a wide-ranging network of bio-chemical signals.

These biochemical signals result in extensive downstream effects including angiogenesis (development of blood vessels, as indicated by some of biological processes), collagen biosynthesis and fibroblast propagation^48^. The epithelial transition to mesenchymal cells occurs during tissue/organ fibrosis as a result of injury and ‘PI3K-Akt signaling pathway’ mediates the process of this transition^49^. Some of the up-regulated genes included insulin-like growth factor-I (IGF-I), semaphorin 3C (SEMA3C), tenascin-C (TNC), transforming growth factor-β (TGFβ2), and genes for the families of fibroblast growth factors, integrins and collagens.

IGF-I and SEMA3C have a developmental role in tissue repair, regulation of epithelial junction and epithelium morphogenesis^50,51^. TNC is an extracellular-matrix (ECM) glycoprotein that is highly expressed during embryogenesis and re-appears in response to tissue injury, during wound healing, and with Crohn’s disease and ulcerative colitis^52–56^. TGF-β promotes the expression of TNC so both are linked to induction of tissue repair mechanisms with diarrhoea^52^. TGF-β is also important in the healing of intestinal epithelium, in preventing susceptibility to injury^57^ and in the promotion of tissue fibrosis. In the high-diarrhoea sheep, we observed several upregulated genes in the FGF family, including FGF5, FGF10, FGF11 and FGFR1, all of which play roles in tissue repair, homeostasis and organ development, including the gastrointestinal tract^58^. FGFR1 is a receptor for fibroblast growth factor 2 (FGF2), a factor that interacts with IL-17 in the repair of damaged intestinal epithelium^59^.

The organization of the ECM also involves genes that transcribe for collagen, including COL5A1, COL6A3, COL8A2 and COL27A1, also known for their role in tissue healing^60^ and are activated as a result of tissue damage. Similarly, the integrin family helps maintain the integrity of the ECM by promoting ECM adhesion, one of the most important biological process identified in our GO analysis^61^. Overall, therefore, we have shown that the fibroblast, integrin and collagen family genes are activated in the high-diarrhoea sheep, strongly indicating ongoing fibrosis. As they establish in the gut mucosa, the helminths promote fibrosis and are thus able evade the immune response mounted by the host. Fibrosis can also lead to alterations in the way the immune system responds, as seen in cystic fibrosis, where there is a decrease in the expression of MHC class II molecules^62^, a similar scenario observed in high diarrhoea animals (decreased expression of Ovar-DRB1 and DQA).

On the other hand, most of the genes that were down-regulated with high diarrhoea, compared low diarrhoea, are directly or indirectly involved in the immune response, the regulation of immune response and immune tolerance: ‘antigen processing and presentation’, ‘antigen processing and presentation of peptide or polysaccharide antigen via MHC class II’, ‘intestinal immune network for IgA production’, ‘primary immunodeficiency’ and ‘immune response’. The processing, conversion and presentation of helminth antigen through MHC-II molecules is critical in mounting a protective immune response so down-regulating this process can make the sheep susceptible to infection, leading to helminth establishment and possibly diarrhoea^63,64^. Immunoglobulin-A (IgA) has been associated with the length and fecundity of helminths^65^ so down-regulation of ‘intestinal immune network for IgA production’ suggests a failure to arrest helminth growth and reproduction, allowing establishment of a successful population and ultimately gut damage leading to diarrhoea.

Other DEGs that were down-regulated in the high-diarrhoea group include Ovar-DRB1, DQA, TREM2, TFF3, ITLN2, CD74, CCL5, and CCR3. Ovar-DRB1, a Major Histocompatibility Complex class II (MHC-II) gene, is widely associated with resistance to nematodes in sheep^31,66,67^ and underpins presentation of antigenic molecules to T-cells, an integral component of the adaptive immune response^68^. MHC-II molecules, present on antigen presenting cells, tend to present parasitic antigens to T-helper type 2 (TH2) cells to initiate the antibody-producing adaptive immune response that is required to control helminths^14,69^. In addition to its role in resistance to roundworms, Ovar-DRB1 has been associated with resistance to tapeworms in Chinese Merino sheep^70^. Carriers of Ovar-DRB1 show reductions in duodenal contractile force and FEC^30,31,66^ so down-regulation of Ovar-DRB1 may increase duodenal contractions^71^ and thus explain our high-diarrhoea phenotype. DQA is similar to Ovar-DRB1 in that it is a MHC-II molecule that plays an important role in adaptive immunity by presenting processed antigen to T-cells^72,73^.

The triggering receptor expressed on myeloid cells (TREM2), also found on intestinal macrophages and lamina-propria DCs, has inflammatory activities, so down-regulation of TREM2 would lead to immune suppression by decreasing pro-inflammatory cytokine production and T-cell activation^74,75^. Trefoil factor 3 (TFF3) and sheep intelectin-2 (ITLN2) normally help the host to combat helminths by altering luminal mucus^76,77^, but here we see a down-regulation of these genes with the high-diarrhoea phenotype, indicating a reduction of two important Th2-cytokines, IL-4 and IL-13^78^. Cluster of differentiation (CD74) is a class-II cell surface receptor for the macrophage migration inhibition factor (MIF) that is expressed by antigen-presenting cells (APCs) and epithelial cells^79^. Binding of MIF to CD74 induces intracellular cell survival cascades^80^ and regeneration of intestinal epithelial cells^81^ leading to healing of the mucosa.

Chemokine ligand 5 (CCL5; also known as RANTES), an important chemokine found in eosinophil granules, is required for normal T-cell function and for recruiting other lymphocytes, such as monocytes and T-cells, to the site of infection/inflammation^82^. CCL5 interacts with CCR3 (a chemokine receptor) leading to a Th2-polarised immune response^83^. Thus, the down-regulation of CCL5 in high-diarrhoea sheep complements an overall suppression of the immune response to helminth infection.

In addition to suppression of the immune system, the sub-network analysis also identified a set of down-regulated genes related to immune tolerance, a way of limiting the immune response after it has been initiated in response to a foreign antigen/pathogen. It is important for the response to subside, or at least be kept under control, so the host does not develop autoimmunity/hypersensitivity^84^. One such check is mediated by Indoleamine2,3-Dioxygenase (IDO), an important rate-limiting enzyme that catabolises tryptophan^85,86^ to kynurenine. This process is crucial in modulating an adaptive immune response by inhibiting effector T-cell proliferation and by diverting the immune response from effector T-cells to regulatory T-cells^87^. In inflammatory bowel syndrome (IBD) and murine models of colitis, the severity of disease is restrained by induction of IDO^88^. In general, therefore, IDO suppresses immune responses promoting immune tolerance and preventing autoimmunity^89–91^. The suppression of immune tolerance in the high-diarrhoea sheep could lead to a potential inflammatory/hypersensitive immune response but, overall, the down-regulation of the immune response outweighs the possibility that ‘suppressed immune tolerance’ could lead to a pathological hypersensitive immune response.

## Conclusion

We have been able to identify pathways and biological process in the immune system that respond to helminth infection and might explain why some sheep are susceptible to the development of diarrhoea while others are not. Importantly, it has become clear that diarrhoea in the diarrhoea-susceptible sheep is not caused by an inflammatory/hypersensitive immune response because most of the up-regulated genes are related to repair mechanisms in the duodenum and most of the down-regulated genes are related to different components of immune response. The literature shows that Ovar-DRB1 is associated with helminth resistance and, in our study, down-regulation Ovar-DRB1 was associated with diarrhoea rather than the parasitism itself. The immune tolerance due to tryptophan catabolism by IDO shows how the immune system is regulated and kept in check in low-diarrhoea sheep. Several up-regulated genes in the high-diarrhoea group are related to ECM organization leading to fibrosis, perhaps explaining why these animals have a high-diarrhoea, because the focus of these genes is repair rather than addressing the primary cause. The organization of ECM could also be linked to the maintenance of physical barriers as another way to resist the helminths and maintain tissue integrity. With diarrhoea or helminth infection, injury could change gut morphology for a certain period, perhaps affecting subsequent immune responses. The extent of fibrosis, and its resolution, are yet to be determined but our observations lead us to suggest a ‘remodeled immune’ response and fibrosis that probably provides a ‘helminth hideout’. Some of the biological processes and pathways that we have revealed could explain how diarrhoea develops, but we are still not really sure how some sheep resist diarrhoea while others readily develop it.

## Supplementary Information

Supplementary Figures.

Supplementary Information 2.

Supplementary Information 3.

## Data Availability

The raw sequencing reads generated in this study have been submitted to NCBI Gene Expression Omnibus (GEO) database under the BioProject accession number PRJNA736720.
