# Supplementary material for: Investigating the development of diarrhoea through gene expression analysis in sheep genetically resistant to gastrointestinal helminth infection
Source: Sci Rep. 2022 Feb 9;12:2207. doi: 10.1038/s41598-022-06001-4 (PMC8828848; doi:10.1038/s41598-022-06001-4)
Supplement: Supplementary file 1 — Supplementary Figures. [file 41598_2022_6001_MOESM1_ESM.docx]

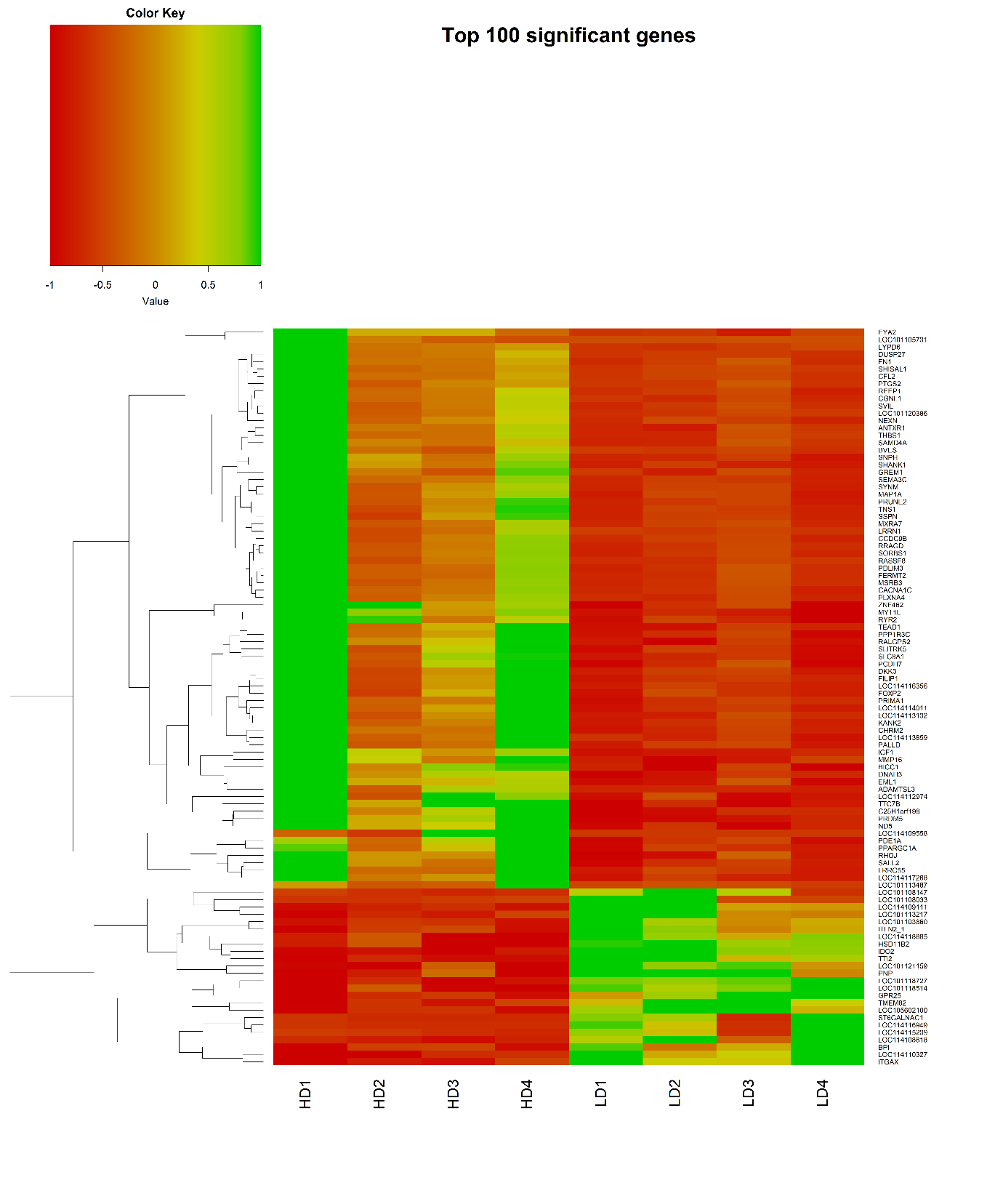


Supplementary Figure S1

A heatmap illustrating the top 100 most significant differentially expressed genes in diarrhoea-susceptible and diarrhoea-resistant groups.


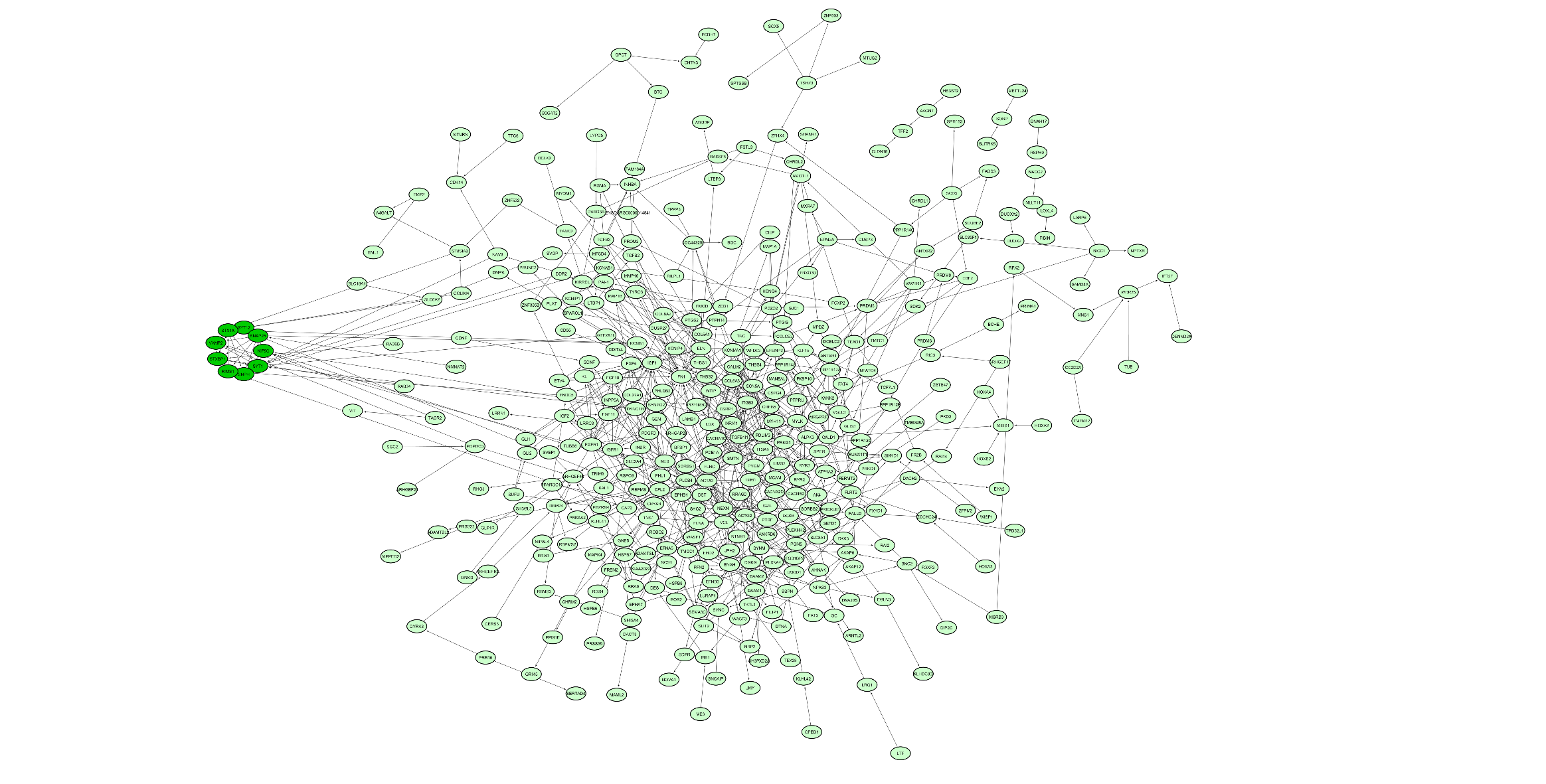


Supplementary Figure S2

Protein-protein interaction (PPI) networks associated with up-regulated DEGs derived from STRING with subnetworks (SN) generated using the Cytoscape ClusterONE plugin. Only one significant SN was associated shown in dark green node (SN1).
